# *In* silico identification of potential key regulatory factors in smoking-induced lung cancer

**DOI:** 10.1186/s12920-017-0284-z

**Published:** 2017-06-07

**Authors:** Salem A. El-aarag, Amal Mahmoud, Medhat H. Hashem, Hatem Abd Elkader, Alaa E. Hemeida, Mahmoud ElHefnawi

**Affiliations:** 1 Bioinformatics Department, Genetic Engineering and Biotechnology Research Institute (GEBRI), University of Sadat City, Sadat City, Egypt; 2 Information Systems Department, Faculty of Computer and Information, Menoufia University, Al Minufiyah, Egypt; 3 Animal biotechnology Department, Genetic Engineering and Biotechnology Research Institute, (GEBRI), University of Sadat City, Sadat City, Egypt; 4 Biomedical Informatics and Chemoinformatics Group, Informatics and Systems Department, National Research Center, Cairo, Egypt; 5 Center of Informatics, Nile university, Sheikh Zayed City, Giza Egypt

**Keywords:** Systems biology, Lung cancer, Network modeling and analysis, Enrichment analysis, Drug targets

## Abstract

**Background:**

Lung cancer is a leading cause of cancer-related death worldwide and is the most commonly diagnosed cancer. Like other cancers, it is a complex and highly heterogeneous disease involving multiple signaling pathways. Identifying potential therapeutic targets is critical for the development of effective treatment strategies.

**Methods:**

We used a systems biology approach to identify potential key regulatory factors in smoking-induced lung cancer. We first identified genes that were differentially expressed between smokers with normal lungs and those with cancerous lungs, then integrated these differentially expressed genes (DEGs) with data from a protein-protein interaction database to build a network model with functional modules for pathway analysis. We also carried out a gene set enrichment analysis of DEG lists using the Kinase Enrichment Analysis (KEA), Protein-Protein Interaction (PPI) hubs, and KEGG (Kyoto Encyclopedia of Genes and Genomes) databases.

**Results:**

Twelve transcription factors were identified as having potential significance in lung cancer (CREB1, NUCKS1, HOXB4, MYCN, MYC, PHF8, TRIM28, WT1, CUX1, CRX, GABP, and TCF3); three of these (CRX, GABP, and TCF) have not been previously implicated in lung carcinogenesis. In addition, 11 kinases were found to be potentially related to lung cancer (MAPK1, IGF1R, RPS6KA1, ATR, MAPK14, MAPK3, MAPK4, MAPK8, PRKCZ, and INSR, and PRKAA1). However, PRKAA1 is reported here for the first time. MEPCE, CDK1, PRKCA, COPS5, GSK3B, BRCA1, EP300, and PIN1 were identified as potential hubs in lung cancer-associated signaling. In addition, we found 18 pathways that were potentially related to lung carcinogenesis, of which 12 (mitogen-activated protein kinase, gonadotropin-releasing hormone, Toll-like receptor, ErbB, and insulin signaling; purine and ether lipid metabolism; adherens junctions; regulation of autophagy; snare interactions in vesicular transport; and cell cycle) have been previously identified.

**Conclusion:**

Our systems-based approach identified potential key molecules in lung carcinogenesis and provides a basis for investigations of tumor development as well as novel drug targets for lung cancer treatment.

## Background

Lung cancer is a complex and highly heterogeneous disease involving multiple signaling pathways [1]. It is the leading cause of cancer mortality in men and the second leading cause in women worldwide [2]. Small cell lung carcinoma (SCLC) and non-small cell lung carcinoma (NSCLC) are the main types of lung cancer. The latter represents 80% of lung cancer cases and can be subclassified as squamous cell carcinoma, adenocarcinoma, or large cell carcinoma [3, 4]. Smoking is a major contributor to lung cancer development, being responsible for about 90% of cases [4]. Cigarette smoke induces inflammation and causes oxidative stress and genetic and epigenetic abnormalities that alter gene expression throughout the respiratory tract [5, 6]. Differences in gene expression in large airway epithelial cells between non-smokers and smokers have been analyzed by DNA microarray to determine the effect of smoking on the transcriptome [7]. Tobacco smoke was found to cause lung cancer by inducing of IκB kinase β- and c-Jun N-terminal kinase 1-dependent inflammation [8].

Spira et al. [9] compared gene expression data from smokers with (*n* = 60) and without (*n* = 69) lung cancer. Using a weighted-voting algorithm, these authors identified an 80-biomarker probe set that distinguished these two populations with an accuracy of 83% when validated using an independent test set (*n* = 52). They selected the 40 most frequently upregulated and downregulated probe sets by internal cross-validation [9]. However, this method—which uses only gene expression profiles—does not provide an integrated view. To address this issue, another study established a set of 40 biomarkers with potentially important roles in lung carcinogenesis using a network-based approach that integrated microarray gene expression profiles and information on protein-protein interactions (PPIs) [10]. Network-based approaches in the study of human disease can elucidate the genes and pathways involved as well as biomarkers and potential drug targets [11]. Network reconstruction and gene-set enrichment analysis (GSEA) have been used to mine masses of complex data obtained from genomics, proteomics, phosphoproteomics, and transcriptomics studies and organize them into a coherent global framework [12].

In this study, gene expression data from smokers with lung cancer and those without lung cancer were analyzed using a systems biology approach that included network-based and enrichment analysis of differentially expressed genes (DEGs) between normal and cancerous lung to identify potential key factors contributing to lung cancer progression.

## Methods

**Fig. 1:**
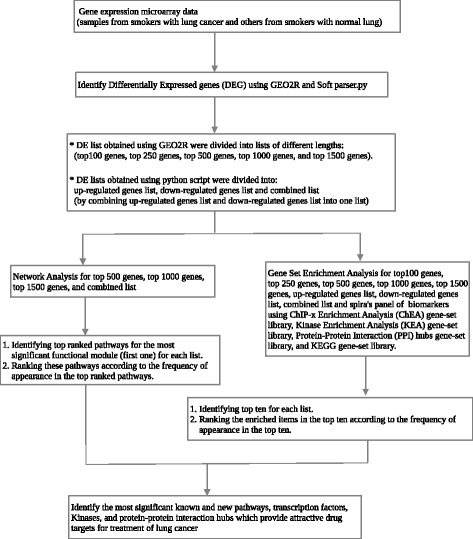
Flow chart of systems biology approach to identify key regulatory factors in smoking-lung cancer

Our strategy for identifying potential key regulatory factors in smoking-induced lung cancer is shown in Fig. 1. We first identified genes that were differentially expressed between smokers with normal lungs and those with cancerous lungs. We then integrated DEG data with information obtained from a PPI database to build a network model, which we used to identify functional modules and relevant signaling pathways. Finally, we carried out a GSEA of DEGs using ChIP-x Enrichment Analysis (ChEA), Kinase Enrichment Analysis (KEA), Protein-Protein Interaction (PPI) hubs, and KEGG (Kyoto Encyclopedia of Genes and Genomes) gene-set libraries

### Dataset

Gene expression data was obtained from Gene Expression Omnibus database (DataSet Record GDS2771). Spira et al. [9] used Affymetrix HG-U133A microarrays to perform gene-expression profiling of large airway epithelial cells obtained by bronchoscopy of current and former smokers. Each individual was followed after bronchoscopy until a final diagnosis of either presence or absence of lung cancer [9]. Data included in our analysis were from smokers with lung cancer (*n* = 97) and those with normal lungs (*n* = 90).

### Identifying DEGs

GEO2R and Soft parser.py analysis tools were used to identify DEGs. GEO2R uses Linear Models for Microarray Analysis R packages for background correction and normalization of gene expression data. Benjamini-Hochberg false discovery rate algorithm was used to correct for multiple testing in GEO2R [13].

### Integration of DEGs with PPI database and pathway analysis using atBioNet

atBioNet identifies statistically significant functional modules using a fast network-clustering algorithm called Structural Clustering Algorithm for Networks (SCAN). atBioNet interface is connected to KEGG pathway information to allow assessment of biological functions of the modules through enrichment analysis. Each module has a pathway summary ranked according to Fisher’s exact test *P* value; The pathway with the lowest *P* value is considered as the most significant [14]. Only large DEG lists such as the combined list, GEO2R lists (top 500, 1000, and 1500 genes) were used as input lists for atBioNet, which was adjusted using the most stringent options that were not appropriate for smaller DEG lists. Of the three options for node addition, we selected the most stringent [“add only nodes directly connected to at least two input nodes (more stringent)”]. From two human PPI databases, we selected a smaller and more robust database (K2 Human Subset Database) obtained by the integration of seven original databases using K-votes approach [14].

### GSEA using Enrichr

Enrichr includes 35 gene-set libraries, some of which are unique to this web server [15]. We used ChEA, KEA, PPI hubs, and KEGG gene-set libraries in this study. Enrichment was computed with the z-score method which outperformed the standard Fisher’s exact test and a combined scoring method that computed a combined *P* value from Fisher’s exact test and the z-score of the deviation from the expected rank [15]. As the enrichment analysis is sensitive to input genes of variable lengths, different input list sizes (from nine lists) were included to ensure that our conclusion was reliable as we concentrated on enriched items with higher overlap in these lists. Up- and downregulated gene lists, the combined list, GEO2R lists of different lengths (top 100, 250, 500, 1000, and 1500 genes) and the spira’s panel of an 80-gene biomarker [9] were used as separate input lists for Enrichr. The Spira’s panel of an 80-gene biomarker [9] was included as an independent list to enrich our study with the results of the enrichment analysis for this valuable list.

## Results

### Pathway analysis of DEG lists with PPI databases

**Fig. 2:**
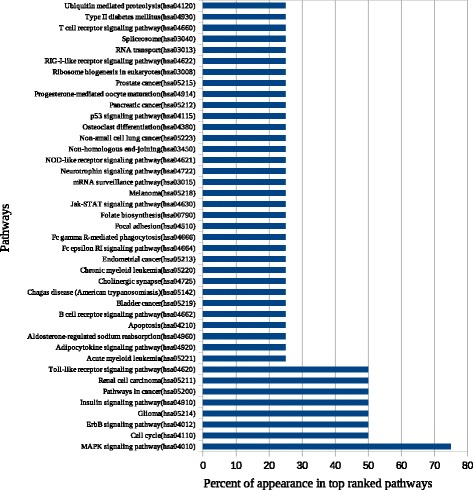
Top ranked pathways using atBioNet. The figure illustrates that MAPK signaling pathway is the most significant pathway related to lung cancer in smokers. Also, cell cycle, ErbB signaling pathway, glioma, insulin signaling pathway, pathways in cancer, renal cell carcinoma, and Toll-like receptor signaling pathway, and ether lipid metabolism are highly related to lung cancer

We identified DEGs using GEO2R and Python script analysis tools. With GEO2R, the top DEGs were divided into different lists according to length (top 100, 250, 500, 1000, and 1500 genes). With the Python script tool, DEG lists were divided into lists of genes that were up- and downregulated as well as a list combining both of these groups. The combined list and GEO2R output lists of different lengths (500, 1000, and 1500 genes) were used as input lists for atBioNet. Top-ranked pathways for the most significant functional modules generated for each list were ranked according to the frequency percent of appearance in the top-ranked pathways lists (Fig. 2).

### Enrichment Analysis

Up- and downregulated gene lists, the combined list, GEO2R lists of different lengths (top 100, 250, 500, 1000, and 1500 genes), and Spira’s 80-gene panel [9] were used as separate input lists for Enrichr.

**Fig. 3:**
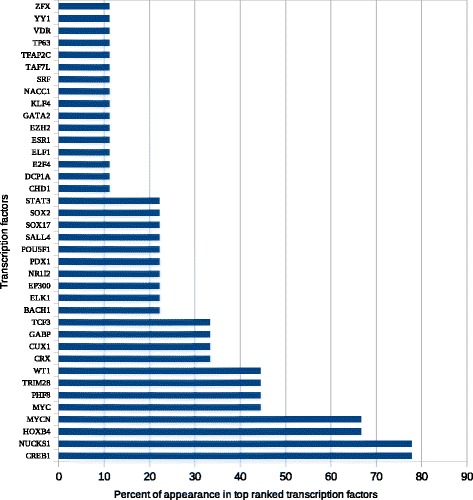
Transcription factors enrichment analysis using ChEA gene-set library. The transcription factors CREB1, NUCKS1, HOXB4, and MYCN frequently appeared as top-ranked transcription factors. CRX, TCF3, and GABP were predicted as novel putative transcription factors in lung cancer

**Fig. 4:**
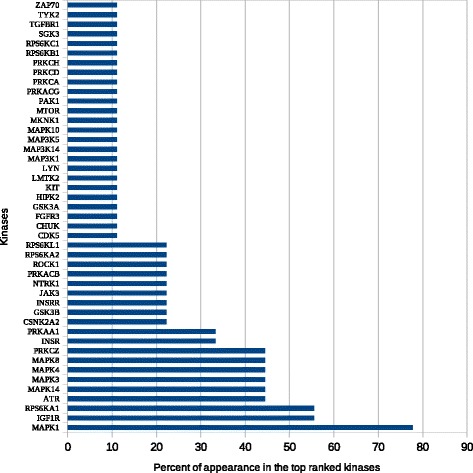
Kinases enrichment analysis using KEA gene-set library. MAPK1, IGFIR, and RPS6KA1 were the top-ranked kinases with frequency percentages of about 80% for MAPK1 and 55% for each of IGFIR and RPS6KA1. PRKAA1 was also predicted as a new putative kinase in lung cancer

**Fig. 5:**
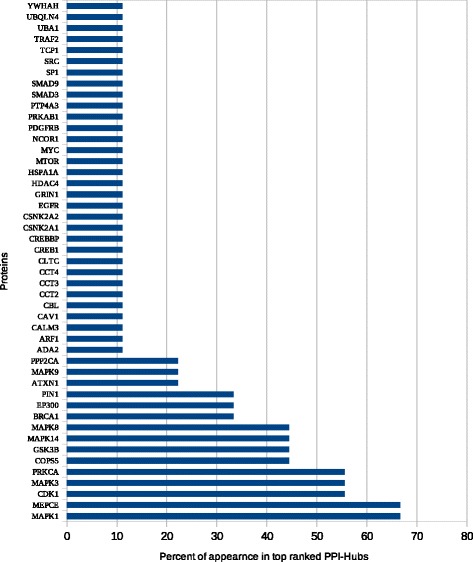
Enrichment analysis of PPI hubs. MAPK1, MEPCE, CDK1, MAPK3, and PRKCA frequently appeared in top 10 PPI hubs in about 70% of input lists

**Fig. 6:**
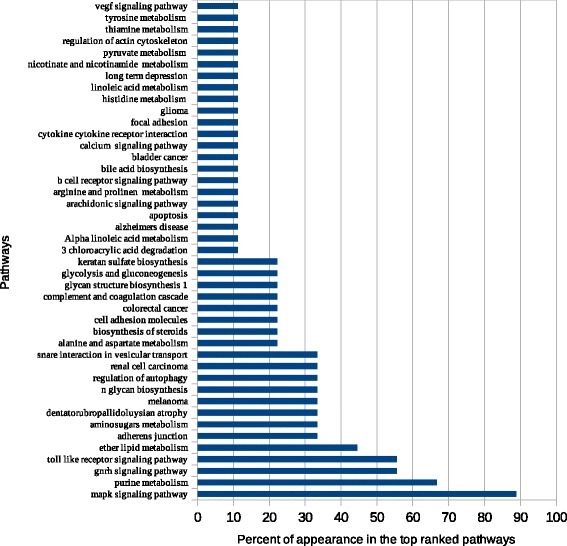
Enrichment analysis of pathways using KEGG gene-set library. Pathway enrichment analysis revealed MAPK signaling pathway to be in the top ten in about 90% of input lists. Purin metabolism, GnRH signaling pathway, Toll-like receptor signaling pathway, and ether lipid metabolism were highly related to lung cancer. Amino sugar metabolism and N-glycan biosynthesis were predicted to be dysregulated pathways in lung cancer

Top-ranked enriched data generated for each list were ranked according to the frequency percent of their appearance in the top ten (Figs. 3, 4, 5 and 6). The transcription factors CREB1, NUCKS1, HOXB4, and MYCN frequently appeared as top-ranked transcription factors. CRX, TCF3, and GABP were predicted as novel putative transcription factors in lung cancer (Fig. 3). Enrichment analysis of kinases showed that MAPK1, IGFIR, and RPS6KA1 were the top-ranked kinases with frequency percentages of about 80% for MAPK1 and 55% for each of IGFIR and RPS6KA1. PRKAA1 was also predicted as a new putative kinase in lung cancer (Fig. 4). MAPK1, MEPCE, CDK1, MAPK3, and PRKCA frequently appeared in top 10 PPI hubs in about 70% of input lists (Fig. 5). Pathway enrichment analysis revealed MAPK signaling to be in the top ten in about 90% of input lists. Purine and ether lipid metabolism and gonadotropin-releasing hormone (GnRH) and Toll-like receptor (TLR) signaling pathways were highly related to lung cancer. Amino sugar metabolism and N-glycan biosynthesis were predicted to be dysregulated pathways in lung cancer (Fig. 6).

## Discussion

Cancer is a complex disease and carcinogenesis in humans is a multistep process that transforms normal Cells into malignant derivatives so that investigation of the carcinogenesis from the systems perspective is inevitable [10]. Many studies have identified potential biomarkers for lung cancer using integrative approaches. Liu et al. [16] identified twelve proteins [p-CREB(Ser133), p-ERK1/2(Thr202/Tyr204), Cyclin B1, p-PDK1(Ser241), CDK4, CDK2, HSP90, CDC2p34, β-catenin, EGFR, XIAP and PCNA] which can distinguish normal and tumor samples with 97% accuracy and four proteins (CDK4, HSP90, p-CREB and CREB) which can be used to calculate the risk score of each individual patient with NSCLC to predict survival. This study identified the top six canonical pathways dysregulated in NSCLC—i.e., ATM signaling, PI3K/AKT signaling, p53 signaling, PTEN signaling, ERK/MAPK signaling, and EGF signaling. Byers et al. [17] found that SCLCs showed lower levels of several receptor tyrosine kinases and decreased activation of phosphoinositide 3-kinase (PI3K) and Ras/mitogen-activated protein (MAP)/extracellular signal-regulated kinase (ERK) kinase (MEK) pathways but significantly increased levels of E2F1-regulated factors including enhancer of zeste homolog 2 (EZH2), thymidylate synthase, apoptosis mediators, and DNA repair proteins. These authors also found that PARP1 1—a DNA repair protein and E2F1 co-activator—was highly expressed at the mRNA and protein levels in SCLCs. In addition, a smoking-associated six-gene signature for predicting lung cancer risk and probability of survival has been established [4].

In this study, nine top-ranked transcription factors (CREB1, NUCKS1, HOXB4, MYCN, MYC, PHF8, TRIM28, WT1, CUX1) (Fig. 2) were found to be significant in lung cancer (Table 1) [18–42], and three (CRX, GABP, and TCF3) were newly identified as potentially significant transcription factors in smoking-induced lung cancer. CRX (Cone-rod homeobox protein) has been proposed as a sensitive and specific clinical marker and potential therapeutic target in retinoblastoma and pineoblastoma [43], and is essential for growth of tumor cells with photoreceptor differentiation [44]. GABP (GA-binding protein) selectively activates the mutant TERT promoter in cancer which in turn enables cells to escape apoptosis, fundamental steps in the initiation of human cancer [45]. A TCF3-PBX1 fusion gene has been detected in adenocarcinoma in situ [46].

**Table 1:** Predicted novel and known Therapeutic transcription factors

Symbol	Description	Literature evidence
CREB1	Cyclic AMP-responsive element-binding protein 1	[18–21]
NUCKS1	Nuclear ubiquitous casein and cyclin-dependent kinase substrate 1A, P1	[22, 23]
HOXB4	Homeobox protein Hox-B4, HOX2F	[24, 25]
MYCN	N-myc proto-oncogene protein	[26–28]
MYC	Myc proto-oncogene protein	[26, 29]
PHF8	Histone lysine demethylase PHF8	[30, 31]
TRIM28	Transcription intermediary factor 1-beta (TIF1-beta)	[32–34]
WT1	Wilms tumor protein	[35–38]
CRX	Cone-rod homeobox protein	New
CUX1	Homeobox protein cut-like 1	[39–42]
GABP	GA-binding protein	New
TCF3	Transcription factor E2-alpha	New

The top ten kinases in the present study (MAPK1, IGF1R, RPS6KA1, ATR, MAPK14, MAPK3, MAPK4, MAPK8, PRKCZ, INSR) have been previously identified (Table 2) [3, 47–63]. However, this is the first report of PRKAA1 as a significant factor in lung carcinogenesis induced by smoking. PRKAA1 (5′-AMP-activated protein kinase catalytic subunit alpha-1) mediates autophagy during differentiation of human monocytesis and can potentially serve as a therapeutic target in chronic myelomonocytic leukemia [64].

**Table 2:** Predicted novel and known therapeutic kinases

Symbol	Description	Literature evidence		
MAPK1	Mitogen-activated protein kinase 1 or ERK-2	[47, 48]		
IGF1R	Insulin-like growth factor 1 receptor	[49–52]		
RPS6KA1	Ribosomal protein S6 kinase alpha-1 or RSK-1	[53, 54]		
ATR	Serine/threonine-protein kinase	[55]		
MAPK14	Mitogen-activated protein kinase 14 or MAP kinase p38 alpha	[56, 57]		
MAPK3	Mitogen-activated protein kinase 3 or Extracellular signal-regulated kinase 1 (ERK-1)	[58, 59]		
MAPK4	Mitogen-activated protein kinase 4 or Extracellular signal-regulated kinase 4 (ERK-4)	[60]		
MAPK8	Mitogen-activated protein kinase 8 or Stress-activated protein kinase JNK1 or c-Jun N-terminal kinase 1	[61, 62]		
PRKCZ	Protein kinase C zeta type	[63]		
INSR	Insulin receptor	[3]		
PRKAA1	5′-AMP-activated protein kinase catalytic subunit alpha-1	New		

Eight proteins were significantly related to lung carcinogenesis—i.e., MEPCE, CDK1, PRKCA, COPS5, GSK3B, BRCA1, EP300, and PIN1 (Table 3) [3, 65–76]. Network analysis (Fig. 2) and enrichment analysis (Fig. 6) showed that MAPK signaling is the most significant pathway related to lung cancer in smokers. Both approaches identified that MAPK, TLR, and renal cell carcinoma signaling pathways a as being important in smoking-induced lung cancer. In addition, purine and ether lipid metabolism; GnRH, ErbB, and insulin signaling; adherens junctions; regulation of autophagy; snare interaction in vesicular transport; and cell cycle were also found to play important roles (Table 4) [77–91], whereas six pathways (aminosugars metabolism, dentatorubropallidoluysian atrophy, melanoma, N-glycan biosynthesis, renal cell carcinoma, and glioma) were predicted here for the first time as being significant pathways in smoking-induced lung cancer.

**Table 3:** Predicted PPI- hubs

Symbol	Description	Literature evidence
MEPCE	7SK snRNA methylphosphate capping enzyme or Bicoid-interacting protein 3 homolog (Bin3 homolog)	[3]
CDK1	Cyclin-dependent kinase 1	[65]
PRKCA	Protein kinase C alpha type or PKC-A	[66]
COPS5	COP9 signalosome complex subunit 5	[67, 68]
GSK3B	Glycogen synthase kinase-3 beta	[69]
BRCA1	Breast cancer type 1 susceptibility protein	[70, 71]
EP300	Histone acetyltransferase p300 or p300 HAT	[72, 73]
PIN1	Peptidyl-prolyl cis-trans isomerase NIMA-interacting 1	[74, 76]

**Table 4:** Predicted novel and known dysregulated pathways in lung cancer

Pathways	References
MAPK signaling pathway	[77]
Purine metabolism	[78]
GnRH signaling pathway	[79]
Toll-like receptor signaling pathway	[80, 81]
Ether lipid metabolism	[82]
Adherens junction	[83]
Aminosugars metabolism	New
Dentatorubropallidoluysian atrophy	New
Melanoma	
N-glycan biosynthesis	New
Regulation of autophagy	[84]
Renal cell carcinoma	
Snare interaction in vesicular transport	[85, 86]
Cell cycle	[87]
ErbB signaling pathway	[88]
Glioma	
Insulin signaling pathway	[89–91]
Pathways in cancer	

Increased glycolysis is a metabolic hallmark of cancer [92]. Cancer cells can reprogram glucose metabolism and hence, energy production by limiting energy metabolism to glycolysis, resulting in an aerobic glycolytic state [93]. Cancer cell metabolism is aimed at increasing biomass (e.g., nucleotides, amino acids, and lipids) to produce a new cell [94]. Melanoma, renal cell carcinoma, and glioma have all been found to be potentially related to lung cancer. Bean et al. [95] identified that targeting MET may be therapeutic target for treatment of a gefitinib/erlotinib-resistant lung tumor cell line with acquired MET amplification. Moreover, dysregulation of MET signaling has been associated with both sporadic and inherited forms of human papillary renal carcinomas [96]. The five components of the dentatorubropallidoluysian atrophy signaling pathway have been shown to have predictive power for breast cancer prognosis [97].

## Conclusion

In this study, we used a systems-based approach to identify potential key molecules and pathways contributing to lung cancer progression among smokers. Three transcription factors (CRX, GABP, and TCF3) and one kinase (PRKAA1) were predicted here for the first time as being important in lung carcinogenesis. In addition, various intracellular signaling pathways and metabolic and other cellular processes were found to be closely related to lung cancer. Our findings provide new insight into the mechanisms of lung cancer development as well as potential new drug targets for disease treatment.

## Abbreviations

ChEA: ChIP-x enrichment analysis
DEG: Differentially expressed genes
KEA: Kinase enrichment analysis
KEGG: Kyoto encyclopedia of genes and genomes
PPI: Protein-protein interaction
